# Once upon a crush story: transforming relationships and sexuality education with a post-qualitative art-ful praxis

**DOI:** 10.1080/14681811.2022.2090915

**Published:** 2022-07-22

**Authors:** EJ Renold, Victoria Timperley

**Affiliations:** School of Social Sciences, Cardiff University, Wales, UK

**Keywords:** Creative, sexuality education, professional learning, queer, posthuman

## Abstract

This paper shares a rhizomatic unfolding of how a creative, post-qualitative praxis for becoming adventurous in the field of Relationships and Sexuality Education (RSE) can unfold in a conducive policy and practice context (Wales, UK). Specifically, we focus on the making and mattering of what we call ‘Crush-Cards’. These are a suite of illustrated data calling-cards designed to re-animate research findings and stay close to the ways in which children and young people are entangled in, and navigate their way through, complex human and more-than-human gender and sexuality assemblages. Each section progressively provides a glimpse at how our art-ful rhizomatic praxis has evolved and how the resource and emergent CRUSHing pedagogy is becoming resourceful in unexpected ways.

## Introduction: becoming adventurous in making research matter

This paper takes inspiration from Kathleen Quinlivan’s (Gilbert 2022) spirit for adventure in sexuality education research by drawing on the word’s Latin roots, *adventurus* – ‘a thing about to happen’, and its 14^th^ century meaning as a ‘dangerous undertaking’. Developing a speculative praxis for becoming adventurous in the schizoid, risk-averse/risk-saturated field of school-based sexuality education, will always be fraught and dangerous. This is because doing sexuality education Otherwise, as Quinlivan (2018, 145) has argued for, often ‘involves the formidable and destabilising challenge of letting go of what adults have already deemed young people need to know about sexuality and relationships’. Our praxis is in dialogue with and contributes to how sexuality education researchers are experimenting with different ways to register and communicate how children and young people are navigating the increasingly visible, uneven, contracting and expanding worlds of gender and sexuality across education policy and practice terrains (e.g. Allen and Rasmussen 2018; Alldred and Fox 2019; Allen 2020; Atkinson et al. 2022; Ollis et al. 2022).

We write from the UK at a time when each of the four nation-states is significantly revising their school-based Relationships and Sex/uality Education (RSE) provision. In such times, experimenting with what else RSE research can do has become increasingly urgent, yet increasingly risky, especially when researchers, like EJ are deeply embedded and becoming more and more implicated with what unfolds (see https://bit.ly/371dNDe). The more public our interventions and inventions in this field become, the more such adventures become fuel for toxic social media debate, Twitter trolling and hate-mail. Nevertheless, we persist. As Quinlivan (2018, 146) has advocated, there is a desperate need to ‘hold possibilities for cultivating pedagogical dispositions which can enable both teachers and researchers to move away from linear and hierarchical approaches to sexuality education in schools and cultivate possibilities for making it more relevant and meaningful for young people’.

Over the last 10 years, EJ (see Renold and Ivinson 2022), and more recently, Vicky (Timperley 2020), have been developing a *post-qualitative, art-ful* praxis. The ‘post-qualitative’ (St. Pierre 2011) in our praxis challenges what counts as research, how it unfolds and how it matters (Taylor, Quinn, and Franklin-Phipps 2020). The art in our praxis draws specifically on Erin Manning’s (2020, p.22) notion of art as the transformational process of coming to know differently through creativity as a *way* to notice the always already queerness of the world and imagine Otherwise. Like much post-qualitative arts-based youth research (see Lupton and Leahy 2021), art-as-way is a process of creating ethical, political research-engagement-activist spaces for surfacing what matters (see Renold and Ivinson 2022). For ourselves this has involved co-producing research, resources, events and pedagogies with others (e.g. other academics, artists, teachers, youth workers, third sector workers, civil servants, politicians, and young people).

In the spirit of Kathleen’s provocation to nurture the “rich possibilities for sexuality education as the ‘art of the possible’ (Quinlivan 2018, 169), our contribution is thus to share one such art-ful post-qualitative adventure through the making and mattering of a collection of data calling-cards that we call ‘Crush-Cards’. We crafted these cards as an invitation to educators to ‘empirically attune’ (Stewart 2014) to the ways children and young people are entangled in and navigate their way through, some complex human and more-than-human gender and sexuality assemblages. What started out as an inventive resource for a specific intervention with in-service teachers, continues to become resourceful. It was also a journey that Kathleen was a part of^1^ and continues to participate in and inform through the rich legacy of her scholarship and practice.

## Why ‘crush cards’? Attuning to what matters

Over the last seven years we have been coproducing gender and sexuality education resources in the field of school-based Relationships and Sex/uality Education (RSE). These are resources designed to enable practitioners to engage more directly with ‘what matters’ to children and young people. This work is often fraught with risk, moral panic and an often-wilful distancing, neglect or incapacity to register the complexity of children and young people lives in this area (Gilbert 2021). Following the turbulent ‘success’ of EJ’s Welsh Government sponsored arts-activist AGENDA resource (www.agendaonline.co.uk), EJ has since been directly involved in sparking and sustaining a risky, radical overhaul of a national curriculum in Wales (https://www.cardiff.ac.uk/research/impact-and-innovation/research-impact/transforming-relationships-and-sex-education-in-wales-england-and-internationally). This has included the development of an explicitly LGBTQ+ inclusive, rights and equity informed, cross-curricular statutory RSE (from 3–16), which is attempting to champion a grass-roots co-construction of the RSE curriculum with teachers, students, other stake-holders and academics (Welsh Government 2022).

It was during the early curriculum-making workshops, as teachers drafted the ‘what matters’ learning objectives for a formal RSE curriculum that EJ found themselves repeatedly sharing research stories with teachers to unsettle their various assumptions of children and young people’s lived experience on matters of gender and sexuality. Unplanned, but in response to this ‘problem space’ (Lury 2021) EJ offered to run two, two-day intensive workshops with primary, secondary and special school teachers. The lofty and impossible aim was to introduce the potentially transformative vision of RSE in Wales and stay close to ‘what matters’. These workshops developed into a bespoke creative professional learning programme (PLP), with Ester McGeeney and Leanne Coll preparing teachers for the new statutory RSE in 2022 (see Renold, Ashton, and McGeeney 2021). And with each cohort (2018–19, 2019–20, 2020–21, 2021–2022) the programme expands, and resources are co-produced. The making of the crush-cards has become one of the key resources, and has evolved into one of the ‘tiny thousand methodologies’ (Lather 2013, 643, citing Deleuze) in a longitudinal creative post-qualitative praxis that has been becoming resourceful ever since, and is slowly beginning to ‘reinvigorate sexuality educators’ everyday work in schools’ (Quinlivan 2018, 169).

**Figure 1.**
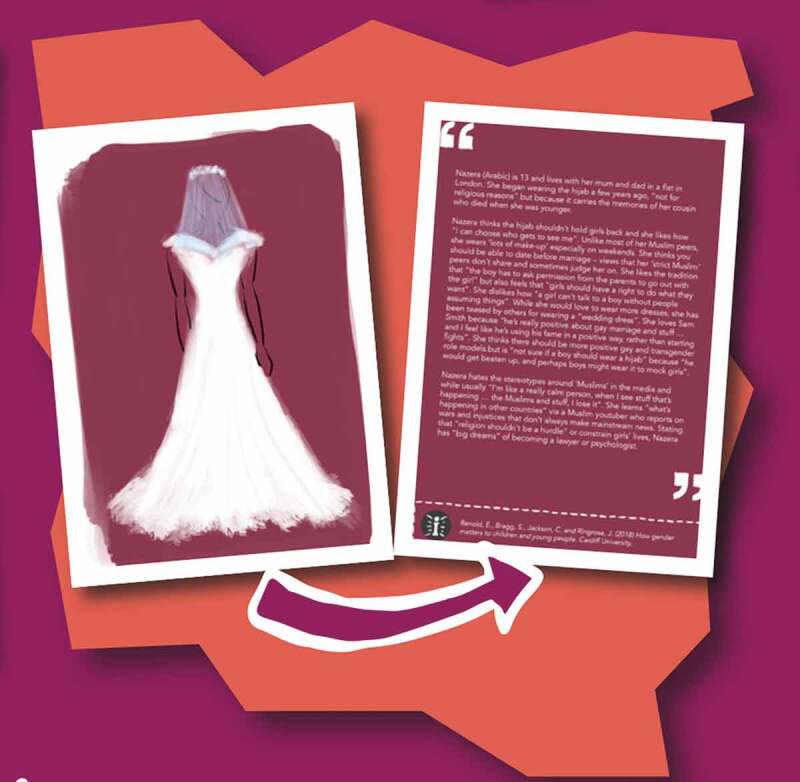
Crush-card example.

The cards were originally designed to invite teachers to ‘empirically attune’ (Stewart 2014) to the ways in which children and young people are differently positioned and entangled in dynamic and complex human and more-than-human gender and sexuality assemblages, where change and transformation demands an ethical-political understanding of a more-than-human agency. Each card contains a bold image on one side and research data in the form of direct quotes from children and young people (vox pops) or ethnographic narratives (vignettes) on the other (see Figure 1).

Much of the crush-card data is drawn from a range of collaborative and individual research projects^2^ spanning 10 years, mostly in Wales. At the bottom of each card is an information icon that links directly to the report, paper, chapter or book. This is an explicit intervention to materialise how ‘research evidence’ matters in an increasingly post-truth world and when much media discourse simplifies, silences or sensationalises the complexity of lived experience. The images are striking and varied. Crucially, they are mostly objects not people. So immediately, you enter the field of RSE via the non-human. The data-image pairings are chosen explicitly to register, unsettle and over-turn assumptions of how the image might (and has) materialised in a young person’s life.

**Figure 2.**
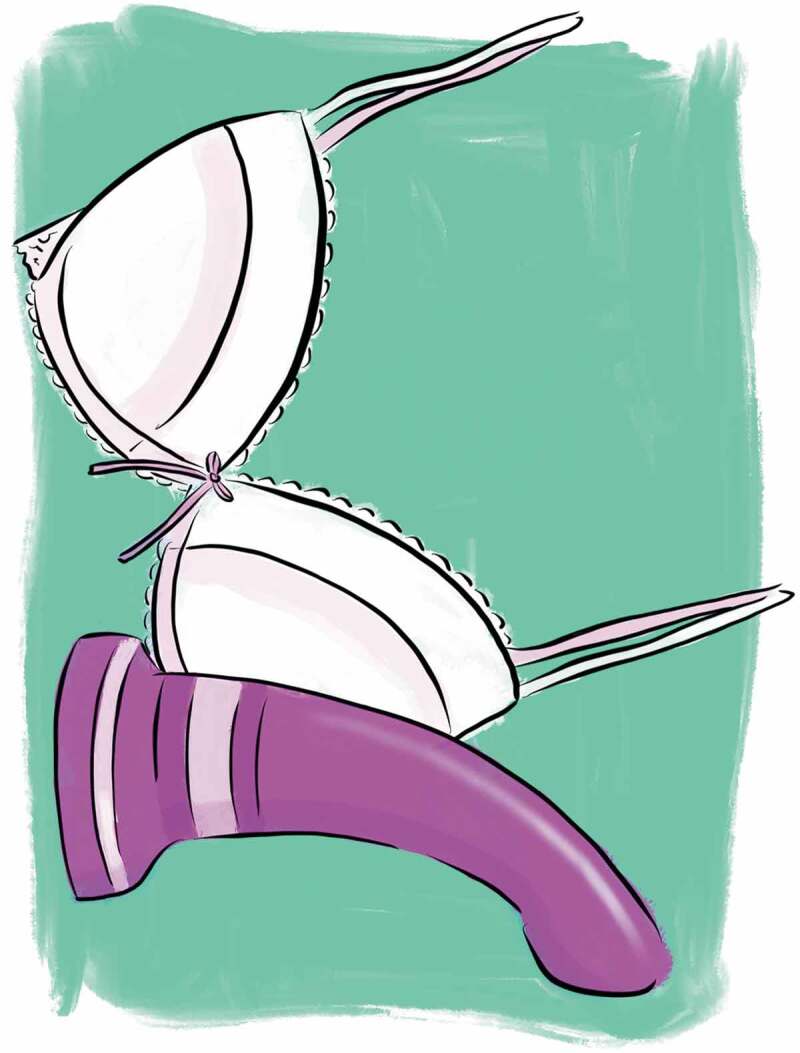
Bra-Dildo crush card.

Half of the images are designed to overturn a potentially recognisable area of RSE (e.g. from sexual health to sexting). Images include a tampon, a dildo, a bra, a mobile phone, red lips. However, the moment of affective, empirical attunement, when the card is turned over, is that the story reorients the reader from what they might have expected. For example, the bra-dildo crush card (see Figure 2) usually generates a lively discussion on ‘premature sexualisation’ and the pornification of sexual learning (Quinlivan 2013b). When the card is turned over, however, the ‘padded bra’ opens up a web of multiple relational meanings.

For the 10-year-old girls that feature on the other side of the card, the padded bra becomes something to hide erect nipples, protect sore developing nipples and enhance the contours of a developing breast to feel older and sexy. Meanwhile the dildo image does not connect to the anticipated ubiquity of sending or receiving dick-pics (Ringrose et al. 2021). Instead, it relates to an ambiguous moment in an arts-based research encounter, when one 12-year-old girl draws a large penis shape onto the body of a girl character she is dressing up to go to the school prom. When quizzed by her friend why the girl might need, ‘one of those?’, she spontaneously responds with, ‘because she’s a girl!’. With no interpretation and no wider assemblage through which to explore this statement, participants are left to wonder, and multiple interpretations abound (see Ashton 2020 for a discursive-affective analysis of teachers’ engagement with this particular crush card at a professional learning conference).

While half of the images connect to recognisable RSE topics, the remainder do not immediately or easily map onto the field of RSE policy or practice. This was an intentional move to invite teachers to reimagine how they might connect to the field of RSE. These images include illustrations of a rifle, a muddy footprint, a mermaid, a squishy cat, a bunny, a birthday card, a picture of Batman, glitter, a towel. The Rifle, for instance, often surfaces talk about war, violent toys and toxic masculinity. However, over-turned, it reveals the story of one neuro-diverse young man’s exploration of his pansexuality that is embraced in his local rural army cadet culture but not mirrored at home and explores the various tensions that play out in and across LGBTQ+ school-based youth groups, family and army life (Kjaran and Sauntson 2019). Finally, some crush-cards unfold the micro-politics of creative survival and resistance in the hidden worlds of children and young people’s lives. These cards connect to the posthuman agency referred to above and more explicitly recognisable stories of young people becoming micro and macro youth activists (Ringrose et al. 2021; Coll et al. 2019).

**Figure 3.**
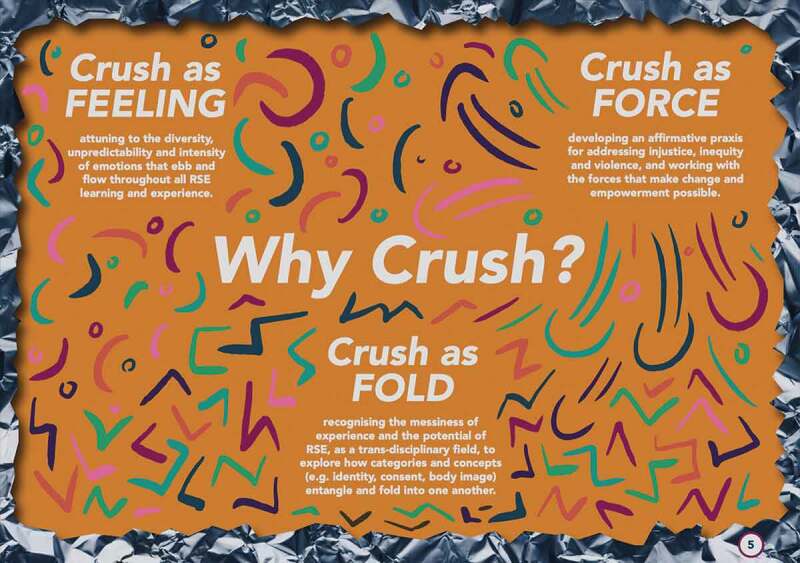
CRUSH as feeling, force and fold.

As a resource and a pedagogy, we have witnessed how the crush cards carry the potential to open up opportunities for educators to empirically attune to the human and more than human folds, feelings and forces of young gender and sexuality assemblages (Figure 3, Renold, Ashton, and McGeeney 2020, 5). Ambivalence and uncertainty are kept on the move as teachers are invited to interact with the cards, sort the cards, guess how the image might feature in a young person’s life. This is a pedagogy that we hope to share in future publications, but as yet remains a practice that has mostly materialised in co-produced resources, such as case-studies and films, rather than academic publications (see Renold, Ashton, and McGeeney 2020). However, what we can share in this truncated piece is a glimpse at this potential, when the crush-cards were adapted by teachers with young people, and then as a research-engagement methodology we describe in as the next section.

## Crush cards on the make

The crush cards have been on the turn, and on the make, ever since our first introduction of them with teachers participating in our professional learning programme in 2018. While this process is only now beginning to be researched, hundreds of teachers have shared and used the cards and the crushing pedagogy with other teachers, school governors and with parents (see Renold, Ashton, and McGeeney 2021). Some teachers have adapted the resource and pedagogy back in schools by sharing just the images, or replacing the images with physical objects with their students. In these instances, we have noticed how young people have been creating stories, sparked from the images or objects alone. Folding this unfolding storying praxis into our most recent research-engagement project (2021–2022) *Unboxing Relationships and Sexuality Education (RSE)* has become particularly generative.

**Figure 4.**
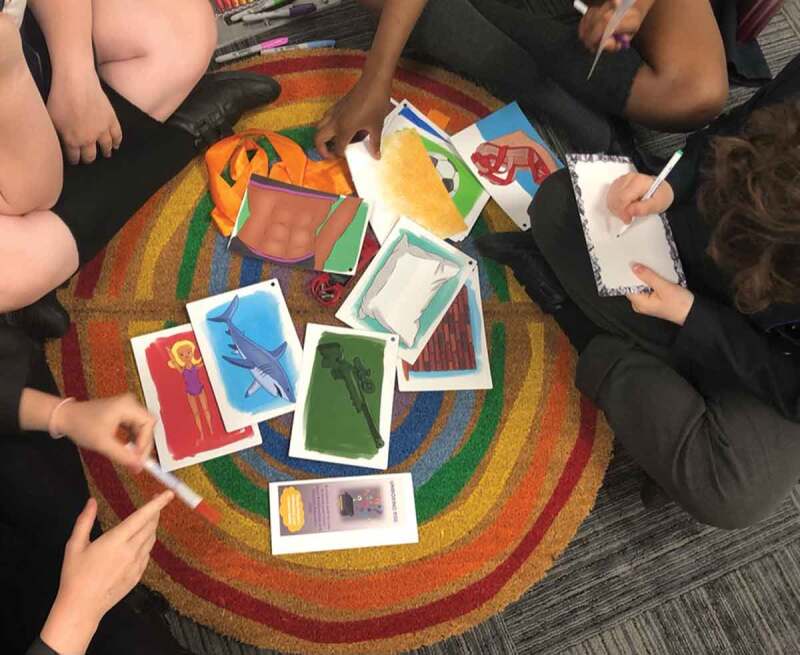
Creating crush-card stories.

*Unboxing RSE* is an exploratory strand of a Wellcome Trust funded public engagement research project.^3^ In the first phase of this project, we invited young people in small friendship groups, to take part in a teacher-researcher facilitated ‘creative audit’. We have been inviting teachers to conduct creative audits since 2018, so this was a funded project that could consolidate and research this process (see Renold, Ashton, and McGeeney 2020, 22–23). The aim of this audit is to invite young people to share what they want to learn, are already learning, and how in the broad area of RSE. The audit uses a range of arts-based activities, including writing on stones, putting messages in jars, pegging up stop/start/continue activist plates and drawing an imagined curriculum. The creative audit begins with young people being invited to interact with a pack of crush-cards (Figure 4). A series of prompts projected onto a large screen titled, ‘Re-image-ining RSE’ invites young people^4^ to ‘spread out the images’, ‘discuss how each image might connect to RSE; ‘guess what the story might be behind the image’; and ‘create your own story using some of the images’ (see the film, Unboxing RSE which captures this process: https://vimeo.com/manage/videos/667900095). While we had envisaged this activity predominantly as a way to platform young people’s own meaning-making and co-create an ethical space that could both hold and play with what surfaced, we had not predicted that the crush-stories would take off and stay with young people in quite the way that they did. Some groups selected images that resonated with them and then created a story line that linked them together. Some young people threw the cards up in the air and created a story sparked by how they landed, others shuffled the cards and created a ‘random story as we go along’.

**Figure 5.**
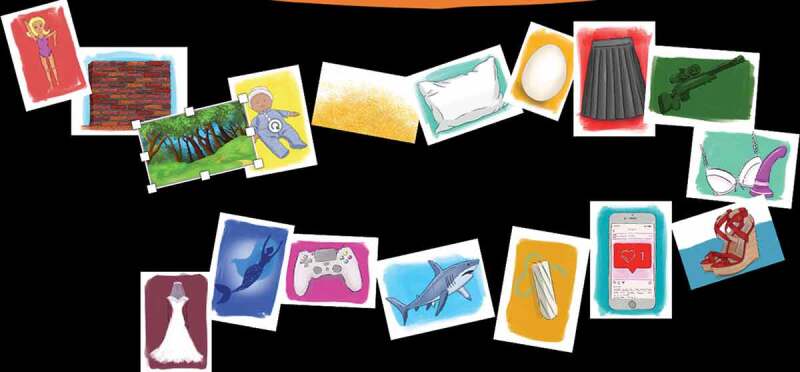
Crush story images.

We conclude this section with the re-telling of a crush-story that one group of girls^5^ from a religiously affiliated school created by spreading out the images and picking up cards that resonated with them. In the follow-up interview after the creative audit, they talked about how they ‘loved’ how they could build ‘fantasy futures’ and ‘make absolutely anything, because it’s just a story … anything can happen’. In under 20 minutes they created their once upon a crush story. It’s a story that captures the potential of a holistic, trans-disciplinary RSE, across areas often deemed taboo, from queer kinship cultures where posthuman desires manifest in mums marrying mermaids, to violent fantasies of blowing up invisible walls. In the spirit of post-qualitative inquiry (see also Renold, Ashton, and McGeeney 2021), we share the story and the images that sparked the story (Figure 5) as a future resource in the making – a story that will be re-animated and take flight in a life beyond this journal article.

## There’s a wall in the forest

There’s a wall*^6^ in the forest*.
The Barbie doll *or the woman is on one side of the wall
on the other side of the wall is her baby*.
The wall is invisible.
It’s covered by magical glitter* which means she can’t see the wall.
The baby is on the pillow * and is protected by an egg*
She needs to go and get the baby because the baby’s crying so is probably hungry.
She uses lots of techniques to get to the other side of the wall.
Under her skirt*, she has a bunch of these cool black widow items like an AK47* and tries to
shoot down the wall
But that doesn’t work.
She uses the wire from her bra*.
But that doesn’t work.
She tries to kick the wall with the heel of her stilettos*
But that doesn’t work.
She uses her phone* to try and call a big strong person, a man, who is a tampon*
(because he’s a straight white male) to come and save her
But that doesn’t work
because the man gets eaten by a shark* and because the tampon swells, the shark dies.
She re-wires an Xbox controller * to make a bomb
that DOES work and blows up the whole thing.
But when she gets through, she sees the mermaid* take the baby.
The mermaid really wants the baby because the mermaid cannot marry a merman because
she’s not allowed and there aren’t any decent ones.
The mum starts chasing the mermaid to try and get her baby back
but when she realises the mermaid wants a baby
because she can’t have her own, she’s like ‘oh that’s sweet’
so they end up getting married* and raise the baby together and live in the forest,
with no wall”.

## CODA: what else can a ‘crush card’ do?

Becoming adventurous in the field of RSE is about making and taking risks for the ethical-political relationalities of an RSE yet to come. Building upon the late Kathleen Quinlivan’s rhizomatic and art-ful approach to transforming school-based sexualities education, we have briefly shared one thread in a conducive policy and practice context of how an artful post-qualitative praxis has unfolded by focusing on the making and mattering of the Crush Cards. These are a suite of illustrated data calling-cards designed to re-animate research findings and stay close to the ways in which children and young people are entangled in, and navigate their way through, complex human and more-than-human gender and sexuality assemblages. As we briefly set out in the section, ‘Why Crush Cards?’, the cards were part of a bespoke professional learning programme designed to experiment with new ways to bring ‘what matters’ to life by offering ‘art-ful encounters’ that facilitate emergent, speculative ways of working (see Renold, Ashton, and McGeeney 2021). The focus in this paper has been to share our rhizomatic journey from the making of the cards in 2018, and how they and we have continued to become resourceful as part of a wider process of co-production to re-matter youth voice and the complexity and diversity of lived lives. Indeed, recent iterations, have included informing national RSE policy and government inquiries into sexual harassment.^7^

While space precludes any deep dive into these complex entanglements, our modest aim in this paper has been to offer a glimpse into an RSE praxis and resource that EJ has called and begun to theorise as CRUSH – because the concepts we use, matter. As Grosz (2011, p. 81) says: ‘The concept is what opens up the thing, objects, process or event – the real – to becoming other, to indeterminate becomings’. Crush is fast becoming a post-qualitative concept that captures our embodied and embedded praxis as sexuality education researchers in Wales. We open this up a little more as we draw the paper to a close. First, ‘crush’ connects to our own desires to ‘do something’ about how research comes to matter and how it might inform (e.g. disrupt, reorient) sexuality education policy and practice. ‘Crush’ also registers and nurtures the generative ‘immaturity’ of an always experimental field of posthuman gender and sexuality studies in childhood and youth (RSE) with a praxis that values the ‘the political value of not being so sure’ and an invitation to ‘risk new relationalities’ (Lather 2013, 640) – a growing up Otherwise (see Atkinson et al. 2022). ‘Crush’ also captures the affective churning or blow that is felt in our gut each time a project trembles and folds, because what surfaces is too troubling and unsettling for different publics to handle (Gilbert 2021). It is a crushing ethical-political praxis that allows us to ‘stay with the trouble’ (Haraway 2016) by making with the trouble (Renold and Ringrose 2019). And it entangles with and builds upon Kathleen Quinlivan’s ‘critical hope’ (Munoz 2009) for how we might make spaces that allow educators to support young people ‘re-vision themselves and their world’ (Quinlivan 2018, 76).

## Notes

1. Significantly, this was an emergent praxis and adventure that Kathleen was about to join us on in the summer of 2019 before her life took a different turn. However, choosing to share the journey of how the crush cards have come to matter, was not just sparked by this potential collaboration. Kathleen first reached out to EJ via a picture postcard, sent from New Zealand to Wales. The card carried a beautiful message in relation to our ‘horse-girl assemblages’ paper and our participatory research with young people in the south Wales valleys (Renold and Ivinson 2013). A connection was formed, and across future conferences and events, shared desires for researching the im/possible (Quinlivan 2013a) more-than-human gender and sexuality activist assemblages in young people’s worlds took shape.

2. They include research by Jessica Ringrose, Sara Bragg, Shiva Zarabadi, Kate Marston, Jen Littleton-Smith, Vicky Timperley and Ester McGeeney.

3. Unboxing RSE is an exploratory strand of a Wellcome Trust funded public engagement research project ‘Engaging Sexual Stories’ (Reference UNS100842). In collaboration, The Open University, UCL London, Cardiff University, and Brook are developing a set of RSE materials from the National Surveys of Sexual Attitudes and Lifestyles (Natsal-3) findings for teachers, young people and the general public. A new suite of ‘crush cards’ has been developed from the first phase of the Unboxing RSE project (https:// agendaonline.co.uk/crush-cards/).

4. We conduced five creative audits with diverse groups of 70+ young people (aged between 13–17) across three high schools (one suburban welsh medium school, one suburban faith school, one semi-rural school).

5. They were a culturally diverse friendship group of (cis-gender) girls, aged between 13–14.

6. Each asterix refers to an image.

7. For example, the cards were pivotal in radically re-routing how Wales’ education inspectorate when EJ was invited to train the inspectors and co-design their methodology. The cards feature on the front cover of the report (see https://www.estyn.gov.wales/thematic-report/we-dont-tell-our-teachers-experiences-peer-peer-sexual-harassment-among-secondary).
